# Medication data harmonization and utilization patterns during pregnancy across claims, electronic health record, and health information exchange data sources

**DOI:** 10.1093/jamiaopen/ooag204

**Published:** 2026-10-26

**Authors:** Jianing Liu, Chienwei Chiang, Xiaofu Liu, Sara K Quinney, Christopher W Bartlett, Shaun Grannis, Lang Li

**Affiliations:** Department of Biomedical Informatics, The Ohio State University, Columbus, OH 43210, United States; Department of Biomedical Informatics, The Ohio State University, Columbus, OH 43210, United States; Department of Biomedical Informatics, The Ohio State University, Columbus, OH 43210, United States; Department of Obstetrics & Gynecology, Indiana University School of Medicine, Indianapolis, IN 46202, United States; Institute for Genomic Medicine, Nationwide Children’s Hospital, Columbus, OH 43205, United States; Data and Analytics, Regenstrief Institute, Indianapolis, IN 46202, United States; Department of Biomedical Informatics, The Ohio State University, Columbus, OH 43210, United States

**Keywords:** pregnancy, drug utilization, electronic health records, semantic interoperability, RxNorm

## Abstract

**Objectives:**

To harmonize pregnancy medication records across heterogeneous real-world data sources and assess the comparability of recorded medication utilization.

**Materials and Methods:**

We analyzed pregnancy medication records from MarketScan administrative claims, the Indiana Network for Patient Care health information exchange, and the Ohio State University Medical Center electronic health record (EHR) system. A deterministic, rule-based pipeline resolved drug names to RxNorm ingredient concepts and mapped them to Anatomical Therapeutic Chemical (ATC) classes. We evaluated mapping coverage and compared utilization using shared ingredient counts, Jaccard similarity across rank thresholds, correlations, and therapeutic-class distributions.

**Results:**

Of 3094 ingredient components across sources, 3085 (99.7%) resolved to RxNorm concepts; 93.9% of resolved components carried an ATC code (93.4%-94.4% by source). The 3 sources shared 759 ingredients, with pairwise Jaccard similarities of 0.68-0.70 across complete lists. However, claims and EHR shared only 2 of their top 10 medications (Jaccard similarity, 0.11), with similarity increasing steadily as more medications were included in the ranked lists. Anatomical Therapeutic Chemical level-2 analysis identified 15 source-enriched classes. Medications identified in only one source had low recorded utilization.

**Discussion:**

High mapping coverage enabled comparison using standardized medication concepts, but substantial utilization differences remained. These patterns were consistent with differences in care settings and medication capture, although their individual contributions could not be isolated.

**Conclusion:**

This framework supports pregnancy medication research by enabling cross-source comparisons and identifying utilization differences requiring source-specific interpretation. Terminology standardization should be accompanied by consideration of how each source generates and captures medication records.

## Introduction

Chronic conditions are common among pregnant individuals,^1^ and medication use during pregnancy is widespread.^2^^,^^3^ Pregnancy-specific evidence remains limited for many medications,^4^ and pregnant people have historically been underrepresented in clinical drug research.^5^ Administrative claims, electronic health records (EHRs), and health information exchanges (HIEs) have, therefore, become essential for studying medication exposure in pregnancy.^6^^,^^7^

These sources differ in how medications are captured and represented.^7^^,^^8^ Claims capture outpatient dispensing but may miss inpatient or delivery medications; EHRs capture medications within a health system but may miss those obtained elsewhere; and HIEs broaden institutional coverage while introducing documentation and terminology heterogeneity. These differences are especially relevant in pregnancy, when exposure is time-sensitive and spans outpatient, inpatient, and obstetric care.^6^^,^^7^ Variation in naming, abbreviations, combination products, salts, and formulations can cause equivalent medications to be counted separately and documentation differences to be mistaken for true differences in use.^9^^,^^10^

The Anatomical Therapeutic Chemical (ATC) system provides a controlled classification for drug ingredients.^11^ Standardizing drug names to ingredient-level concepts and mapping them to such a classification can improve semantic interoperability. We therefore distinguish semantic interoperability, representing heterogeneous records using shared concepts,^12^ from analytic comparability, which we use here to describe whether standardized concepts support comparable utilization analyses. Because data quality and suitability for secondary analysis are task-dependent,^13^ semantic standardization alone cannot establish analytic comparability. Prior studies have described pregnancy medication use within single sources;^14–17^ fewer have compared harmonized utilization across multiple real-world environments representing complementary capture mechanisms.

### Objectives

We applied a medication data harmonization framework across MarketScan claims (MarketScan),^18^ the Indiana Network for Patient Care HIE (INPC),^19^ and the Ohio State University Medical Center EHR (OSUMC).^20^^,^^21^ We aimed to harmonize medications to ingredient-level concepts and ATC classes, compare utilization across sources, and evaluate whether semantic interoperability supports analytic comparability.

## Methods

### Study design, data sources, and cohorts

We conducted a cross-database observational informatics study using MarketScan, INPC, and OSUMC, representing national outpatient pharmacy claims, a multi-institution HIE, and a single academic medical center EHR, respectively. Cohorts included 729 524 patients in MarketScan (2016-2023), 225 684 in INPC (1990-2024), and 398 576 in OSUMC (2015-2025).

Deliveries were identified using source-specific methods and used to anchor the medication-exposure window (Table S1A and B). In MarketScan, deliveries were identified using ICD-10-CM diagnosis codes, supplemented by CPT and ICD-10-PCS procedure codes to improve capture of delivery claims; the earliest date on which any of these codes was recorded was defined as the delivery date. In OSUMC, deliveries were identified using ICD-10-CM delivery diagnosis codes and dated using the corresponding delivery encounter. In INPC, maternal–child pairs were established using maternal–child relationship data, electronic delivery records, and state natality records. The delivery date was obtained from the clinical delivery encounter or birth record. Medication counts were summarized at the distinct-patient level, with each patient counted no more than once per medication regardless of the number of medication records or eligible pregnancies.

Medication exposure was assessed during a fixed predelivery window of 270 days in MarketScan and OSUMC and 9 calendar months (273-276 days) in INPC. In MarketScan, patients were required to maintain continuous enrollment throughout the 270-day predelivery exposure window. The 270-day window approximated 9 months of pregnancy and provided comparable exposure periods across sources when gestational age was unavailable.

### Medication extraction and ingredient-level harmonization

Medications were extracted from the MarketScan GENNME field, the INPC Med Name field, and the Epic SimpleGenericName field in OSUMC via the Lifescale platform (Figure 1).^21^^,^^22^ Because the deidentified extracts provided medication names rather than product-level codes, names were harmonized directly to RxNorm ingredient concepts without transforming the source databases into a common data model.

**Figure 1.**
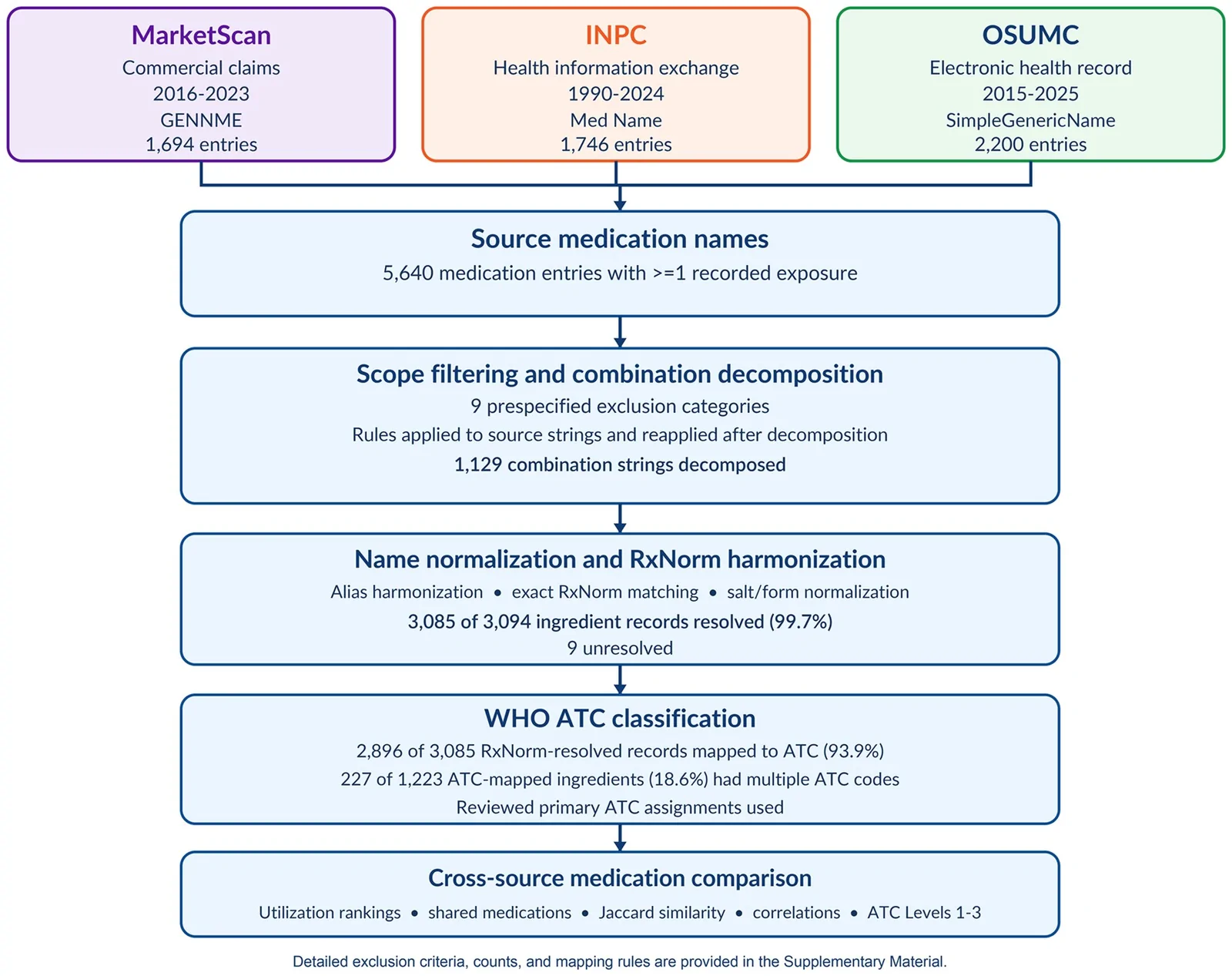
Medication harmonization workflow across MarketScan, INPC, and OSUMC. Medication names were processed using a deterministic, rule-based text normalization pipeline, filtered using prespecified exclusion categories, decomposed into ingredient components when needed, normalized to RxNorm ingredient concepts, and mapped to ATC classes.

Harmonization used a deterministic, rule-based pipeline (Table S2A-C). Approximate string matching was not used to avoid merging similarly spelled but distinct ingredients, such as “inulin” and “insulin.” The pipeline used curated lookup tables, documented regular expressions, and RxNorm lookups.

After Unicode and case normalization, processing proceeded in 3 stages. First, combination products were decomposed into individual ingredients while protecting hyphenated names, stereochemical prefixes, and chemical locants. Formulation descriptors and intravenous vehicle phrases were removed (eg, “CEFAZOLIN IN D5W” to cefazolin), and exclusion rules were reapplied to each component. Second, 244 curated aliases, abbreviations, and truncated forms were standardized, and equivalent entries were merged within each source. Third, names were matched to RxNorm ingredient concepts after removing pharmacopeia qualifiers. For unmatched names, salt, ester, and hydrate descriptors were sequentially removed, with exceptions where these terms were required to preserve ingredient identity. The descriptor list was expanded during review of unresolved source strings.

### Exclusion criteria

Study-scope exclusions were applied to original medication strings before RxNorm resolution and reapplied after combination-product decomposition. Nine categories covered devices, supplies, and kits; irrigation fluids and intravenous vehicles; inert excipients, vehicles, and colorants; nonspecific labels and noningredient fragments; vaccines, toxoids, and diagnostic preparations; nutritional vitamin/mineral supplements and iron products; prenatal and multivitamin bundles; herbals, botanicals and probiotics; and residual nontherapeutic entries (Table S3A).

Retention rules were applied first to preserve 17 therapeutic vitamins or analogs, 31 therapeutic minerals, electrolytes or related salts, and 2 prescription omega-3 products (Table S3B). Oral and parenteral iron products were excluded to avoid preferential retention of formulations more likely captured in inpatient care, and prenatal products were excluded because these multiconstituent products are commonly available over the counter and may be differentially captured across claims, EHR, and HIE data, limiting comparability across sources.

### ATC classification, multiple assignments, and mapping review

Harmonized ingredients were mapped to ATC codes using mappings available in the August 2026 RxNorm release.^11^^,^^23^ Primary analyses used ATC levels 1 and 2. Of 1223 ATC-mapped ingredients, 227 (18.6%) had multiple candidate codes because of differences in therapeutic use, route, or formulation. To avoid double-counting, one primary code was selected using source route or formulation, RxNorm dose form, US product availability, or a prespecified assignment when these were insufficient. The same rules were applied across sources (Table S4).

Harmonization and ATC assignment underwent iterative manual review of unresolved names, resolved ingredients without ATC codes, and ingredients with multiple candidate codes. Errors were corrected through updates to curated tables. Two sensitivity analyses assessed multiple classifications: a level-specific analysis excluded ingredients spanning multiple categories at the ATC level analyzed, and a conservative analysis excluded all ingredients with multiple full ATC codes. These analyses compared within-source category rankings and cross-source utilization correlations, respectively.

### Utilization and comparability measures

Medication utilization was defined as the number of patients with a medication record during the source-specific predelivery window and standardized per 1000 patients. In INPC, privacy-suppressed medication counts reported as <10 were assigned a value of 5 for calculation of standardized utilization rates. At ranked-list thresholds through the complete lists (Table S5), we calculated pairwise and 3-way ingredient overlap and pairwise Jaccard similarity, defined as the number of shared ingredients divided by the union. Pearson, Spearman, and Kendall correlations compared standardized utilization rates among ingredients shared by each source pair.

Medications were grouped at ATC levels 1-3 to calculate category shares of total utilization and cumulative utilization for top level-2 and level-3 categories (Tables S6 and S7). Ingredients observed in only one source were classified as database-specific (Table S8). Level-2 source enrichment was defined as a category’s share in one source minus the mean share in the other 2. Categories were ranked by the margin between the highest and second-highest source shares, and the heatmap displayed those with a margin ≥1.5 percentage points. This threshold was used for visualization rather than statistical inference, and sensitivity analyses were conducted using alternative thresholds (Table S9). Analyses used Python 3.9.21 with pandas, NumPy, SciPy, matplotlib, and seaborn.

## Results

### Harmonization and ATC mapping performance

The harmonized lists included 961 ingredients submitted to RxNorm in MarketScan, 1051 in INPC, and 1082 in OSUMC. Overall, 3085 of 3094 components (99.7%) resolved to RxNorm ingredient concepts, accounting for approximately 100% of exposures; 9 remained unresolved. Of resolved ingredients, 2896 (93.9%) mapped to ATC, representing 97.3% of resolved exposures. Source-specific ATC mapping rates were 94.4% in MarketScan, 93.4% in INPC, and 93.9% in OSUMC (Table 1).

**Table 1.** ATC mapping among RxNorm-resolved ingredients by data source.

Dataset	Total unique RxNorm ingredients	ATC mapped (*n*)	ATC mapped (%)
**MarketScan**	960	906	94.4
**INPC**	1049	980	93.4
**OSUMC**	1076	1010	93.9
**Pooled**	3085	2896	93.9

Pooled counts sum source-specific counts; an ingredient present in more than one source contributes once per source.

### Cross-database comparability and top-ranked overlap

After harmonization, 759 of 1315 distinct ingredients were shared across all 3 sources. Pairwise overlap ranged from 822 to 877 ingredients, with full-list Jaccard similarities of 0.68–0.70 (Figure 2). Database-specific ingredients numbered 67 in MarketScan, 101 in INPC, and 136 in OSUMC.

**Figure 2.**
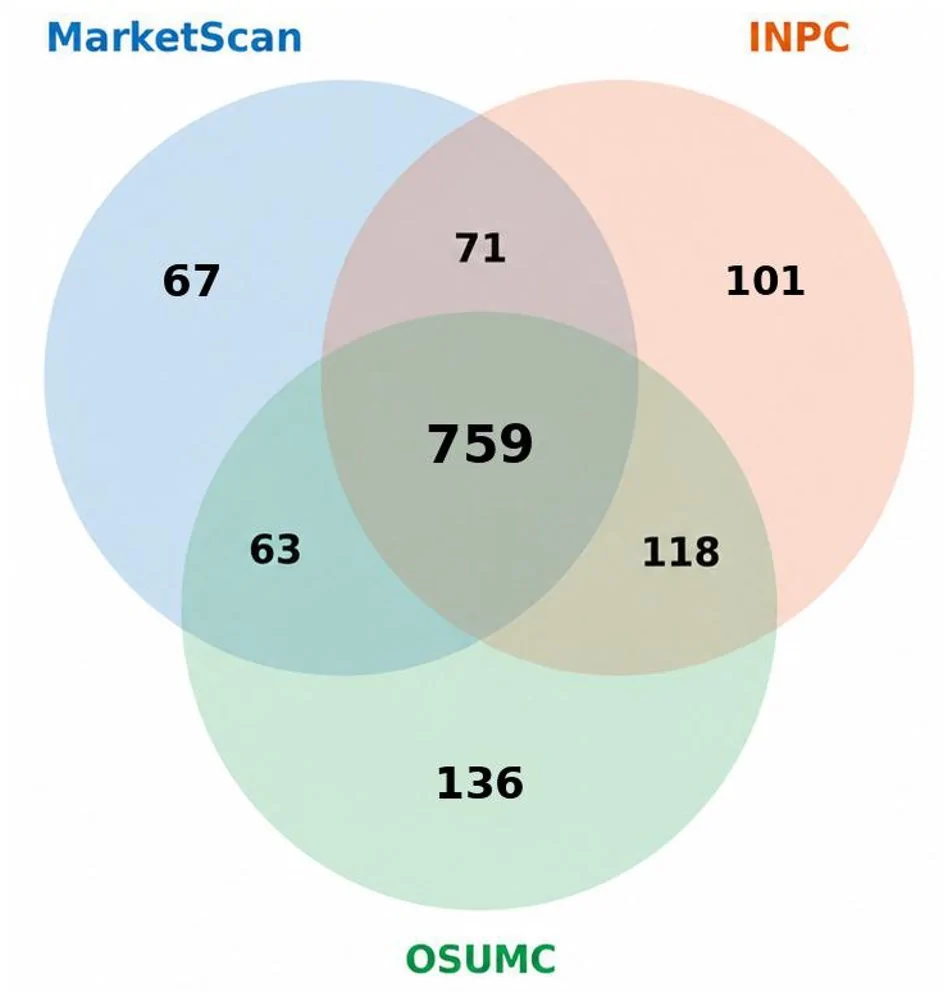
Overlap of harmonized medication ingredients across MarketScan, INPC, and OSUMC. Venn diagram showing medication ingredients unique to and shared among the 3 data sources after ingredient-level harmonization.

Acetaminophen and ondansetron appeared among the top 10 medications in all 3 sources, but the remaining high-frequency medications differed (Table 2). MarketScan was dominated by outpatient and maintenance medications, led by amoxicillin at 131 per 1000 patients; OSUMC by inpatient, procedural, and obstetric medications, led by oxytocin at 317; and INPC showed a mixture of both patterns.

**Table 2.** Top 10 harmonized RxNorm ingredients during pregnancy, standardized per 1000 patients.

Rank	MarketScan	INPC	OSUMC
**1**	Amoxicillin (131)	Acetaminophen (280)	Oxytocin (317)
**2**	Ondansetron (130)	Ondansetron (200)	Lidocaine (316)
**3**	Nitrofurantoin (102)	Promethazine (146)	Sodium chloride (308)
**4**	Azithromycin (96)	Sodium chloride (145)	Acetaminophen (298)
**5**	Progesterone (91)	Metronidazole (143)	Ondansetron (295)
**6**	Pyridoxine (74)	Nitrofurantoin (142)	Docusate (186)
**7**	Acetaminophen (73)	Amoxicillin (130)	Ibuprofen (182)
**8**	Doxylamine (73)	Phenylephrine (129)	Diphenhydramine (177)
**9**	Promethazine (68)	Oxytocin (129)	Sodium citrate (156)
**10**	Metronidazole (68)	Azithromycin (119)	Methylergonovine (141)

Values in parentheses are standardized medication utilization rates per 1000 patients.

Agreement among top-ranked medications was lowest for MarketScan–OSUMC, which shared 2 of the top 10 medications (Jaccard 0.11), compared with 7 for MarketScan–INPC (0.54) and 4 for INPC–OSUMC (0.25). Similarity increased with ranked-list depth (Table 3; Figure S1). By the complete lists, Jaccard similarity reached 0.70 for MarketScan–INPC, 0.68 for MarketScan–OSUMC, and 0.70 for INPC–OSUMC.

**Table 3.** Shared harmonized RxNorm ingredients and Jaccard similarity by rank threshold.

Top *N*	Shared by all 3	MS–INPC Jaccard	MS–OSUMC Jaccard	INPC–OSUMC Jaccard
**10**	2	0.54	0.11	0.25
**100**	44	0.52	0.30	0.50
**200**	118	0.60	0.45	0.66
**450**	306	0.67	0.57	0.67
**800**	554	0.66	0.65	0.67
**All resolved ingredients**	759	0.70	0.68	0.70

Selected thresholds are shown to summarize changes across ranked-list depth; results across all evaluated thresholds are provided in Table S5 and Figure S1.

### Correlation of standardized utilization among shared medications

Utilization correlations varied across source pairs (Table 4). Pearson correlations were 0.75 for MarketScan–INPC, 0.63 for INPC–OSUMC, and 0.27 for MarketScan–OSUMC. Spearman correlations were higher than Pearson correlations in all comparisons, particularly for MarketScan–OSUMC (0.68 vs 0.27). Scatterplots showed corresponding pairwise patterns (Figure 3).

**Figure 3.**
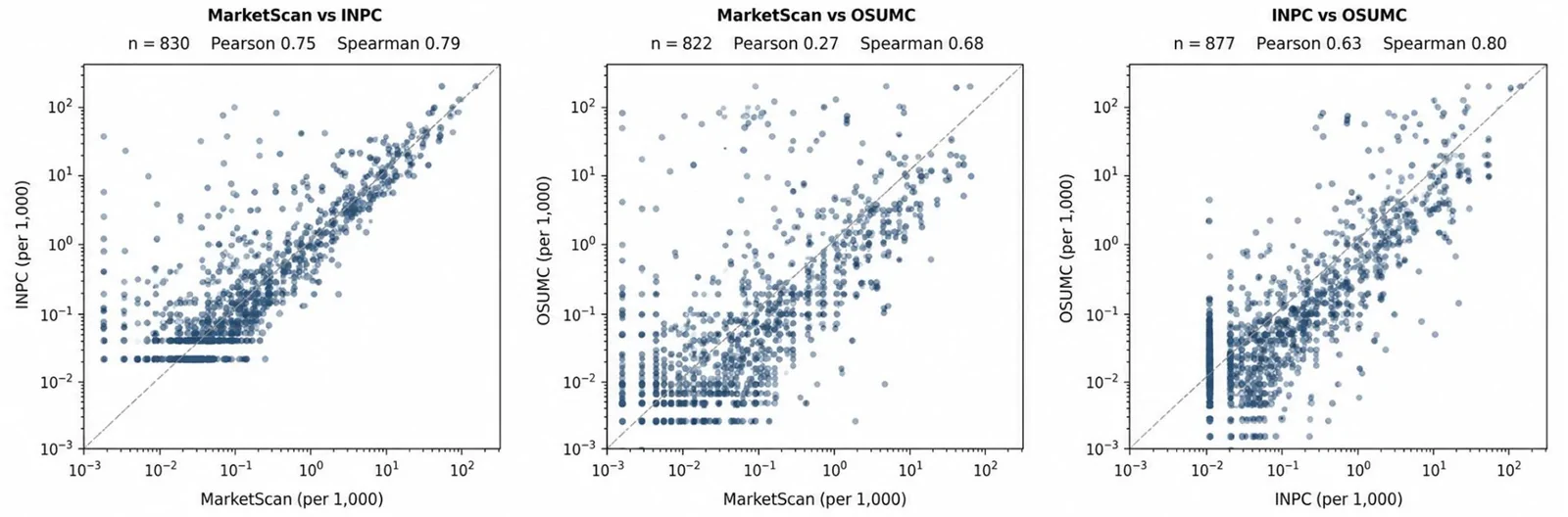
Pairwise comparisons of standardized medication utilization across databases. Scatterplots compare standardized utilization rates per 1000 patients for ingredients shared by each database pair; both axes are logarithmically scaled.

**Table 4.** Pairwise comparisons: shared ingredients and correlation of standardized utilization.

Comparison	Shared ingredients (*n*)	Pearson *r*	Spearman *ρ*	Kendall *τ*
**MarketScan vs INPC**	830	0.75	0.79	0.64
**MarketScan vs OSUMC**	822	0.27	0.68	0.51
**INPC vs OSUMC**	877	0.63	0.80	0.63

Correlations were calculated using standardized utilization rates for ingredients present in both sources of each pair.

### ATC therapeutic distributions and source-specific signatures

Level-1 distributions were broadly similar (Figure 4). Alimentary tract and metabolism drugs accounted for 16.6%, 14.4%, and 23.1% of utilization in MarketScan, INPC, and OSUMC, respectively; nervous system drugs accounted for 12.6%, 21.5%, and 26.0%. Systemic anti-infectives showed greater variation, accounting for 28.9%, 22.4%, and 6.0%, respectively.

**Figure 4.**
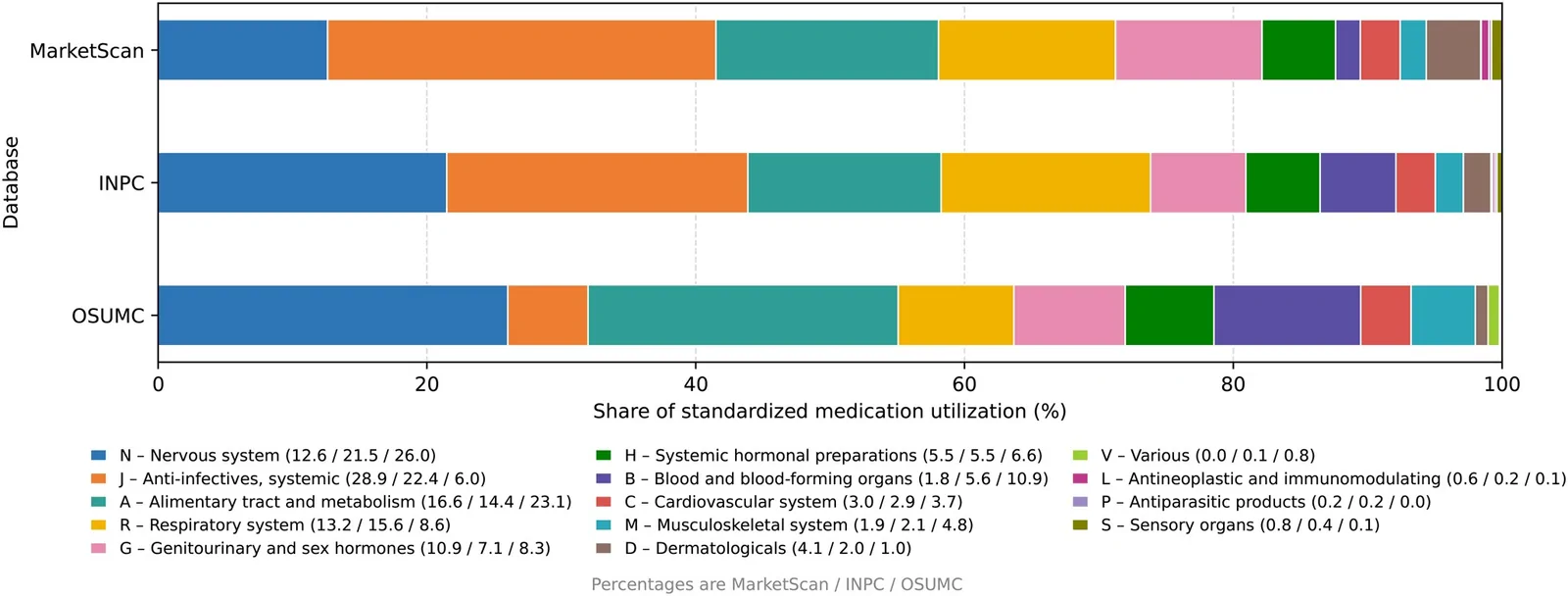
ATC level-1 utilization across databases. Distribution of medication utilization across ATC level-1 classes in MarketScan, INPC, and OSUMC. Percentages represent each class’s share of total standardized medication utilization within a data source.

At level 2, the 20 classes selected across all sources accounted for 72.8%, 81.3%, and 87.2% of utilization in MarketScan, INPC, and OSUMC, respectively (Figure 5). Source-specific top-20 classes accounted for 86.7%, 82.4%, and 89.4%, and the top 30 for 95.1%, 93.3%, and 95.8% (Table S6).

**Figure 5.**
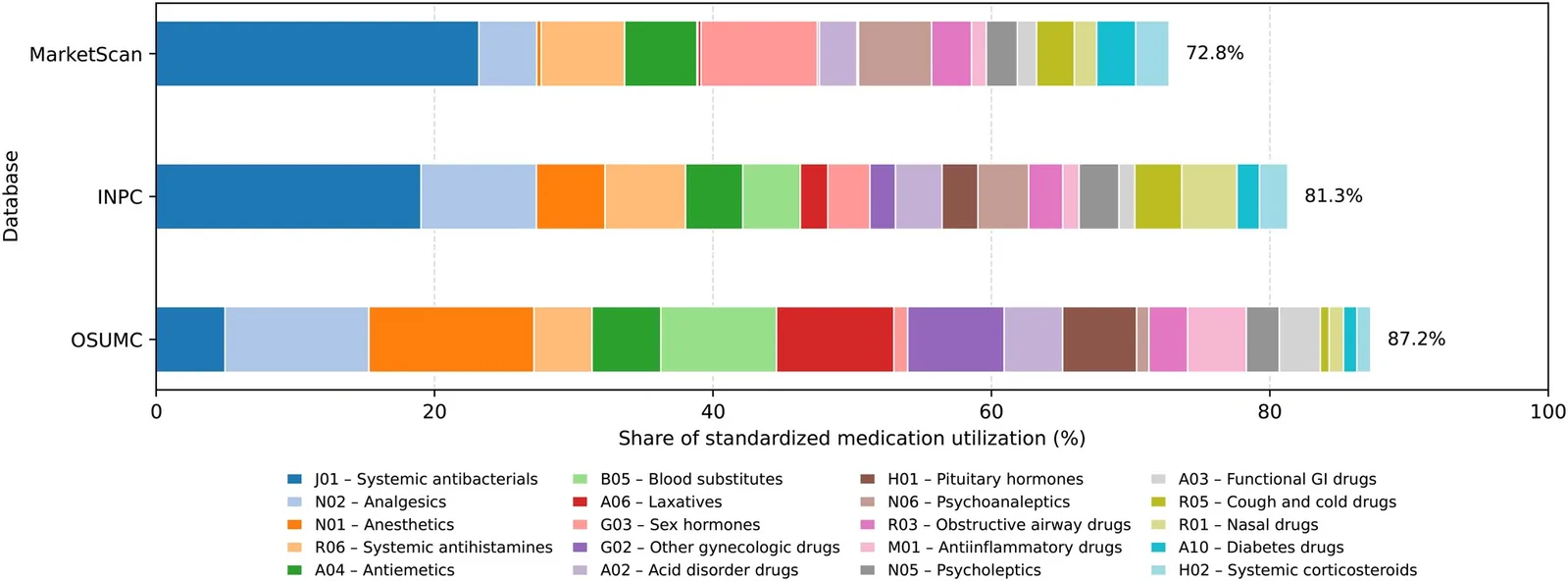
Top 20 ATC level-2 classes across databases. Distribution of standardized medication utilization across the 20 most frequent ATC level-2 classes in the pooled data. Percentages represent each class’s share of total standardized medication utilization within a data source.

The enrichment heatmap identified 15 level-2 categories: 8 enriched in OSUMC, 6 in MarketScan, and 1 in INPC (Figure 6; Table S9A). MarketScan was enriched for outpatient and maintenance classes**–**systemic antibacterials (+11.2 percentage points), sex hormones (+6.3), and psychoanaleptics (+3.0). The OSUMC was enriched for procedural, obstetric, and supportive-care classes**–**anesthetics (+9.2), drugs for constipation (+7.3), other gynecologicals (+5.9), analgesics (+4.1), and pituitary hormones, almost entirely oxytocin given during labor and delivery (+4.0). The INPC’s only enriched class was nasal preparations (+2.6). Sensitivity analyses using alternative enrichment thresholds showed the expected decrease in the number of retained classes as the threshold increased (Table S9B).

**Figure 6.**
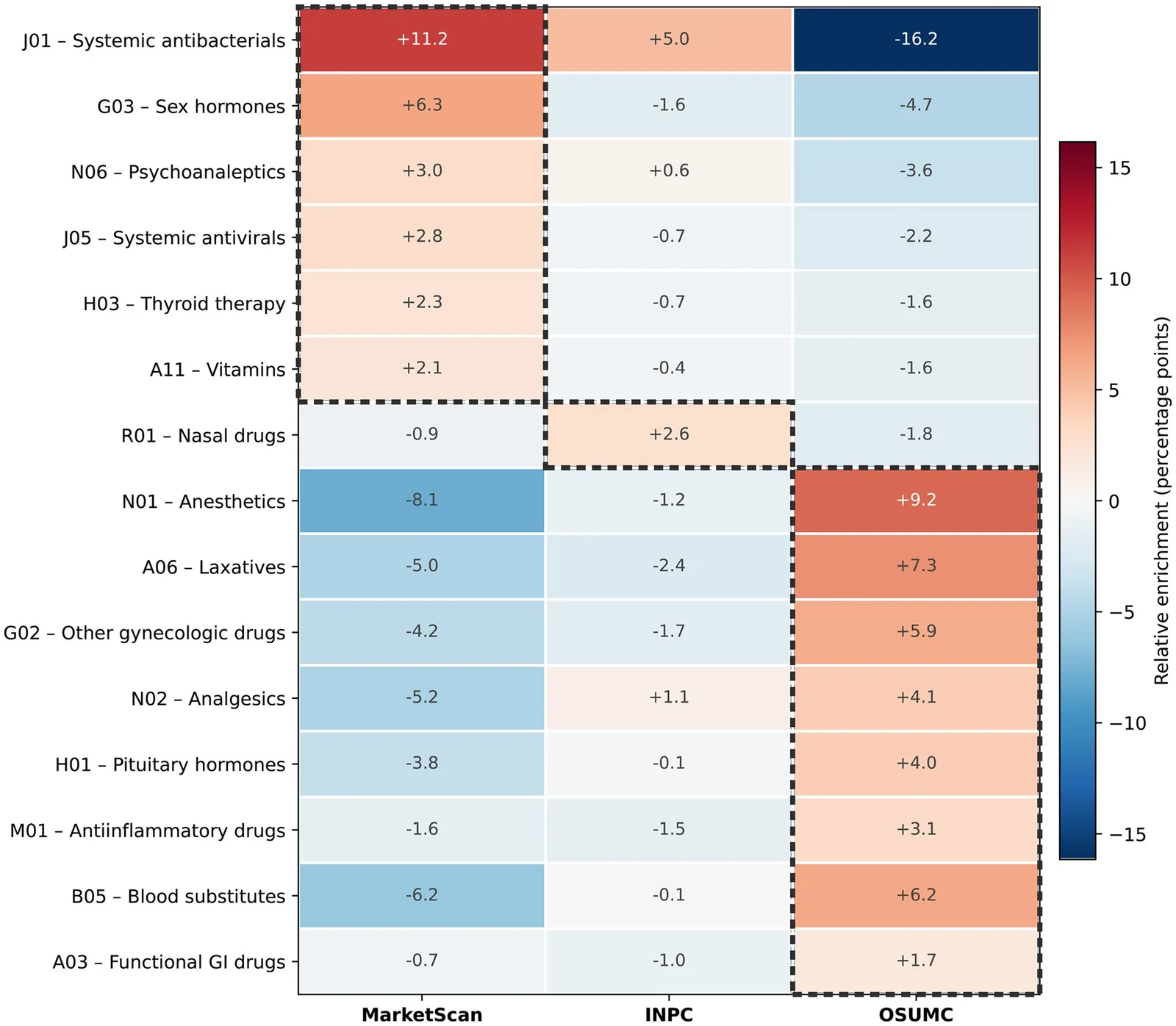
ATC level-2 source enrichment across databases. Heatmap showing enrichment of selected ATC level-2 classes by data source. Values represent the percentage-point difference between each source’s class share and the mean share of the other 2 sources. Classes are displayed when the leading source exceeds the second-highest source by at least 1.5 percentage points; Table S9 reports threshold sensitivity.

### ATC level-3 analysis and sensitivity to ATC assignment

At level 3, the top 20 classes across all sources accounted for 56.3%, 63.3%, and 68.8% of utilization in MarketScan, INPC, and OSUMC, respectively; source-specific top-20 shares were 71.4%, 66.0%, and 79.4% (Figure S2 and Table S7). Spearman correlations of level-3 class shares ranged from 0.71 to 0.89 across source pairs.

Sensitivity analyses showed similar patterns after excluding multiply classified ingredients. Correlations between primary and reduced category rankings remained high at levels 1-3 (Spearman 0.845–0.956), and conservative exclusion of all multiply classified shared ingredients changed pairwise Spearman correlations by no more than 0.031 (Table S4).

### Database-specific medications

A total of 304 ingredients were observed in only one source: 67 in MarketScan, 101 in INPC, and 136 in OSUMC. Of these, 278 had ATC assignments: 62 in MarketScan, 90 in INPC, and 126 in OSUMC (Figure S3). Database-specific medications were individually uncommon, with none reaching 1.0 per 1000 patients. The 50 highest-frequency database-specific ingredients in each source are reported in Table S8.

## Discussion

### Principal findings

Ingredient-level normalization resolved 99.7% of medication components to RxNorm concepts and assigned ATC codes to 93.9% of resolved ingredients, demonstrating that heterogeneous medication text from claims, EHR, and HIE sources can be represented using a common terminology with high coverage. The 3 sources shared a substantial core of 759 ingredients, yet agreement was markedly lower among the medications recorded most frequently. Thus, semantic interoperability did not translate directly into comparable utilization estimates. The major differences across sources were not primarily in whether medications could be represented using common concepts, but in how frequently those shared medications were captured. These findings distinguish 2 related but separate problems in multidatabase medication research: harmonizing what a medication is and understanding what a recorded medication event represents within each data source.

### Semantic harmonization and analytic comparability

Medication names varied across sources through naming conventions, abbreviations, combination products, salt and formulation descriptors, and local documentation practices. Ingredient-level standardization, alias resolution, decomposition, and ATC mapping converted these heterogeneous representations into common analytic units. Prior work has similarly shown that RxNorm-based standardization can normalize heterogeneous medication data for multisource analysis.^9^^,^^10^ The difference between RxNorm resolution and ATC mapping coverage reflects that successful ingredient normalization does not necessarily result in an ATC assignment, as some valid RxNorm ingredients lack a standalone ATC code. Mapping local medication representations to standardized RxNorm concepts is also consistent with medication standardization in common data models such as OMOP,^24^ supporting potential reuse of this harmonization approach in other data environments.

The ranked-overlap analysis demonstrates why terminology harmonization alone is insufficient for cross-database utilization studies. Pairwise Jaccard similarity across the complete medication lists was approximately 0.68–0.70, indicating substantial agreement in the overall medication vocabulary. Agreement among high-frequency medications was much lower, particularly between MarketScan and OSUMC, which shared only 2 of their top 10 medications. Similarity increased progressively as less frequently recorded medications were included. Comparisons involving INPC converged more rapidly, whereas the claims–EHR comparison approached its full-list similarity only at much greater ranked-list depth. These patterns indicate that the databases increasingly converge on a common medication vocabulary in the long tail while differing most strongly in the medications that dominate observed utilization.

The correlation analyses reinforce this distinction. Spearman correlations exceeded Pearson correlations in all 3 database pairs, most prominently for MarketScan vs OSUMC. Thus, sources could show moderate agreement in the relative ordering of shared medications while differing substantially in their recorded utilization rates. This is consistent with prior pregnancy real-world evidence literature showing that medication information is generated and captured differently across administrative claims, EHR, and other healthcare data environments.^6–8^ Outpatient dispensing, prescribing, medication reconciliation, inpatient administration, and delivery-related medication use are therefore not expected to be captured equivalently across sources.

The ATC level-3 analysis showed a similar pattern at a finer pharmacologic level: pairwise correlations remained stronger for MarketScan–INPC and INPC–OSUMC than for MarketScan–OSUMC. The persistence of this pattern across ATC levels suggests that the observed source differences were not solely a consequence of broad therapeutic aggregation.

A potential alternative is to begin harmonization from medication codes rather than names. We used medication names because the available extracts from all 3 sources were text based. Code-based approaches would not eliminate the need for terminology mapping, because medication coding systems differ in granularity and still require cross-terminology mapping to common concepts.^25^ When standardized medication codes are uniformly available, a code-first strategy with text-based fallback may provide stronger provenance. In the present study, however, text-based normalization resolved 99.7% of submitted ingredients, suggesting that the principal remaining challenge was not terminology coverage but interpretation of source-specific medication capture.

### Source-specific medication capture in pregnancy

Previous drug-utilization research has documented substantial variation in medication use during pregnancy across countries and populations, while systematic reviews have noted that differences in study design, exposure definitions, and ascertainment complicate direct comparisons of utilization estimates.^26^^,^^27^ Reviews of pregnancy real-world data similarly emphasize that administrative claims, EHRs, and other data sources differ in available clinical detail and medication-exposure capture.^28^ Our findings extend this literature by showing that substantial cross-source differences remain even after medication names are harmonized to common RxNorm ingredients and ATC classes. The observed therapeutic patterns were compatible with the different roles of the 3 data sources: MarketScan, which primarily reflects outpatient pharmacy dispensing, showed greater representation of systemic antibacterials, sex hormones, and other ambulatory therapies; OSUMC showed stronger representation of anesthetics, obstetric medications, analgesics, and supportive-care drugs, consistent with greater visibility into inpatient and delivery-related care; and INPC generally occupied an intermediate position, consistent with an HIE integrating medication information across participating healthcare settings. These patterns should not be interpreted as evidence of differences in clinical practice alone, because population composition, geography, calendar period, care setting, documentation, and capture mechanisms may also contribute.

Database-specific medications provide a complementary perspective. Although 304 ingredients appeared in only one source, none exceeded 1 per 1000 patients individually. These source-specific ingredients therefore represented a sparse tail of uncommon exposures. Cross-source differences were also evident in the recorded frequencies of medications shared across sources, underscoring that counts of unique medications alone do not characterize differences between databases.

### Implications for pregnancy medication safety and real-world evidence

Claims, EHR, and HIE data therefore provide complementary but nonequivalent views of medication exposure. A recent pregnancy-specific comparison of linked EHR-pharmacy data with administrative claims found high agreement for common medication fills but slightly higher measured prevalence in the linked EHR-pharmacy source.^29^ More broadly, previous studies have shown incomplete agreement between EHR prescribing records and pharmacy claims,^30^ as well as substantial differences in measured opioid exposure between the 2 sources even among the same patients.^31^ These differences partly reflect what each data source records: pharmacy claims primarily reflect dispensing, whereas EHR medication records may reflect prescribing, medication reconciliation, or administration, and HIE data may integrate medication information generated across multiple care settings. Our findings extend this evidence across 3 source types and the broader pregnancy medication landscape, showing that standardized terminology can align medication concepts while recorded utilization remains dependent on the underlying data-generation and capture mechanisms.

This distinction has direct implications for pregnancy medication safety research. Apparent differences in exposure prevalence across databases may reflect differences in healthcare setting, documentation, and medication capture as well as clinical use.^29–31^ The contrast was evident even for widely used medications such as acetaminophen and ondansetron, which ranked among the top 10 in all 3 sources but had substantially different standardized utilization rates. Consequently, cross-database comparisons should not assume that terminology harmonization alone makes exposure estimates interchangeable.

The framework developed here can therefore serve as a diagnostic step before exposure–outcome analyses. Evaluating ingredient overlap, ranked-list agreement, utilization correlations, and therapeutic-class distributions can identify medications and classes for which cross-source estimates are likely to be comparable and those requiring source-specific interpretation. Such evaluation may be particularly important in pregnancy research, where medication-exposure spans outpatient, emergency, inpatient, and delivery settings and no single real-world data source captures all relevant exposure information completely.^28^

### Limitations

Several differences between the data sources should be considered when interpreting these findings. Utilization was standardized per 1000 patients within each source, so differences in cohort size do not directly determine the reported rates. However, the sources differed in population, geography, observation period, and medication capture. The INPC spans a substantially longer period (1990–2024) than MarketScan (2016–2023) and OSUMC (2015–2025). Medication use during pregnancy has changed substantially over time,^3^ and secular changes in prescribing practices, healthcare delivery, coding, and data availability may therefore have influenced recorded utilization. Although INPC showed substantial medication overlap and generally intermediate utilization patterns relative to the other 2 sources, the present study could not isolate temporal effects from differences attributable to database type and capture mechanism.

Medication capture also differed by source. Continuous enrollment in MarketScan establishes insurance coverage but does not guarantee complete medication capture, while EHR and HIE records depend on care delivered or documented within participating systems.^6^^,^^7^ In INPC, privacy-suppressed medication counts reported as <10 were imputed as 5; consequently, utilization estimates for rare medications should be interpreted as approximate. More broadly, individual medication exposures were not independently validated, and correlations were calculated across medications rather than at the patient level. Accordingly, the observed differences may reflect clinical practice, population composition, healthcare setting, data-generation processes, or combinations of these factors. Future analyses using temporally aligned cohorts or linked patient-level data could help distinguish differences in clinical use from differences in data capture.

Approximately 1 in 5 ATC-mapped ingredients had multiple candidate codes, each resolved to a single code against the route or dosage form recorded in the source string, the products RxNorm lists for that ingredient, or the predominant United States product. Sensitivity analyses suggested limited effects on Level-1 distributions and cross-source correlations, although finer-level rankings should be interpreted in light of classification choices. The analysis focused on therapeutic ingredients, retaining selected vitamins, minerals, and electrolytes while excluding nutritional and prenatal supplements and other prespecified products. Findings therefore do not represent all medication and supplement use during pregnancy. Iterative manual review and documented audit trails supported quality control throughout harmonization, although some ambiguity may remain where source names lack sufficient detail to distinguish formulations or clinical uses.

## Conclusion

Medication harmonization achieved near-complete RxNorm resolution across claims, EHR, and HIE pregnancy medication records and revealed a large shared medication vocabulary across the 3 sources. However, high semantic agreement did not imply comparable utilization: differences were greatest among the medications recorded most frequently and persisted across therapeutic classes after harmonization. The principal challenge in cross-database medication research is therefore not only identifying equivalent medication concepts but understanding how each data source generates and captures medication events.

Cross-source pregnancy medication studies should report both terminology harmonization and data provenance, including care setting and medication-capture mechanism. Evaluating overlap and utilization patterns before exposure–outcome analyses can help identify where estimates are likely to be comparable and where source-specific interpretation is required. Harmonization makes cross-database comparison possible; it does not make heterogeneous data sources interchangeable.

## Supplementary Material

ooag204_Supplementary_Data

## Data Availability

Aggregate results are provided in the article and supplementary material. The supplementary material provides the harmonization stages, exclusion and retention criteria, primary ATC assignments, and analysis tables; detailed harmonization dictionaries and audit documentation were supplied for peer review. Additional information may be available from the corresponding author upon reasonable request and subject to required approvals.
