# Supplementary material for: Medication data harmonization and utilization patterns during pregnancy across claims, electronic health record, and health information exchange data sources
Source: JAMIA Open. 2026 Oct 26;9(5):ooag204. doi: 10.1093/jamiaopen/ooag204 (PMC13706625; doi:10.1093/jamiaopen/ooag204)
Supplement: Supplementary file 1 [file ooag204_supplementary_data.pdf]

Supplementary Material

Medication Data Harmonization and Utilization Patterns During Pregnancy Across Claims, Electronic Health Record, and Health Information Exchange Data Sources

Table	Title	Contents
S1	Cohort definitions and medication exposure windows	Panel A: study period, cohort size, delivery identification, delivery-date source, and exposure window by data source. Panel B: delivery-identification codes.
S2	Medication harmonization and terminology mapping	Panel A: harmonization stages. Panel B: reconciliation to the analytic ingredient set. Panel C: RxNorm resolution and ATC assignment by source.
S3	Medication study-scope exclusions and retained therapeutic substances	Panel A: exclusion criteria and counts. Panel B: prespecified therapeutic substances retained under the nutritional-supplement criterion.
S4	Multiple ATC classifications and sensitivity analyses	Summary of multiply classified ingredients, primary-code selection basis, and sensitivity analyses.
S5	Top-ranked medication overlap	Full rank-threshold sweep of shared harmonized ingredients and pairwise Jaccard similarity.
S6	ATC Level-2 cumulative share	Cumulative share of standardized utilization accounted for by the top Level-2 classes within each source.
S7	ATC Level-3 analysis	Top 20 pooled Level-3 classes (as in Supplementary Figure S2), within-source cumulative share, and pairwise correlations.
S8	Database-specific medications	Top 50 harmonized ingredients observed in only one source, ordered by standardized utilization rate.
S9	ATC Level-2 source enrichment and threshold sensitivity	Classes meeting the 1.5-percentage-point margin criterion and sensitivity to alternative thresholds.

Supplementary Figures S1-S3.

S1: Shared medications and Jaccard similarity by rank threshold

S2: Top 20 pooled ATC Level-3 subgroups

S3: ATC Level-1 profile of database-specific medications

Supplementary Table S1. Cohort definitions and medication exposure windows across data sources.

Source-specific cohort characteristics relevant to interpretation of medication utilization.

Panel A. Cohort definitions by data source

Data source	Study period	Pregnancy cohort, n	Delivery identification	Delivery date source	Medication exposure window
MarketScan	2016-2023	729,524	ICD-10-CM diagnosis codes supplemented by CPT and ICD-10-PCS procedure codes (Panel B)	Earliest service date of any qualifying delivery code (diagnosis or procedure)	270 days before delivery
INPC	1990-2024	225,684	Maternal-child linkage using relationship data, electronic delivery records, and state natality records	Clinical delivery encounter or birth record	9 calendar months before delivery
OSUMC	2015-2025	398,576	ICD-10-CM delivery diagnosis codes (Panel B)	Delivery encounter date	270 days before delivery

Panel B. Delivery-identification codes

Category	Code(s)	Code system
Delivery – diagnosis	Z37, Z37.0, Z37.1, Z37.2, Z37.3, Z37.4, Z37.5, Z37.50, Z37.51, Z37.52, Z37.53, Z37.54, Z37.59, Z37.6, Z37.60, Z37.61, Z37.62, Z37.63, Z37.64, Z37.68, Z37.69, Z37.7, Z37.9, O80, O82	ICD-10-CM
Procedure – cesarean delivery	59510, 59514, 59515, 59618, 59620, 59622	CPT
	10D00Z0, 10D00Z1, 10D00Z2	ICD-10-PCS
Procedure – vaginal delivery	59400, 59409, 59410	CPT
Procedure – vaginal delivery after previous cesarean	59610, 59612, 59614	CPT

Supplementary Table S2. Medication harmonization workflow, reconciliation, and terminology mapping.

Harmonization used prespecified deterministic rules; no fuzzy or edit-distance matching was used.

Panel A. Medication harmonization stages

Stage	Step	Purpose	Implementation	Result	Example
1	Lookup normalization	Standardize case, Unicode, punctuation, and whitespace for matching.	Applied to matching keys only; original source strings retained.	-	Vitamin B-12 → vitamin b-12
2	Intravenous vehicle removal	Remove infusion vehicles that are outside the therapeutic-medication scope.	Applied to source strings and decomposed components.	-	CLINDAMYCIN PHOSPHATE IN D5W → clindamycin
3	Formulation and regimen descriptor removal	Remove dosage-form, release, and regimen terms that do not change ingredient identity.	Regular-expression rules.	-	DILTIAZEM HCL ER BEADS → diltiazem
4	Combination decomposition	Split multi-ingredient strings into individual components.	Protected delimiters preserved hyphenated ingredient names and chemical prefixes.	1,129 strings split into 2,000 component rows	PRAMOXINE-HC → pramoxine + hydrocortisone
5	Component salt-abbreviation removal	Remove salt and ester abbreviations within decomposed components.	Rule-based component normalization.	-	Betamethasone Na Phos → betamethasone
6	Alias and shorthand expansion	Expand source-specific abbreviations, contractions, and selected truncations.	Prespecified exact-match alias dictionary; single-word aliases expanded at word boundaries.	244 aliases	APAP → acetaminophen
7	Pharmacopoeia and grade qualifier removal	Remove pharmacopoeia and grade qualifiers that do not change ingredient identity.	Regular-expression rules.	-	Vasopressin (USP) → vasopressin
8	Salt, ester, and hydrate stripping	Remove qualifying descriptors while preserving names in which the descriptor is part of the ingredient identity.	Descriptors stripped one at a time with RxNorm re-checking; protected names were exempt.	93 descriptors; 60 protected names	ESMOLOL HCL → esmolol; sodium chloride protected
9	Truncation repair	Repair source-field truncations only when a single RxNorm ingredient candidate is identified.	Unique-prefix completion; no fuzzy or edit-distance matching.	Unambiguous fragments only	AMOXICILL-CLARITHRO-LANSOPRAZ → amoxicillin (component)
10	RxNorm resolution and ATC assignment	Resolve cleaned ingredients to RxNorm and assign a primary ATC code.	Exact RxNorm lookup with documented bridges/overrides; prespecified primary ATC assignments were used for multiply classified ingredients.	3,085/3,094 resolved to RxNorm (99.7%); 2,896/3,085 RxNorm-resolved ingredients had an ATC code (93.9%).	amoxicillin → RXCUI 723

Panel B. Reconciliation to the analytic ingredient set

Step	n	Note
Recorded source medication rows	5,640	MarketScan, INPC, and OSUMC combined.
Excluded at the source-string stage	849	Study-scope exclusions applied before combination decomposition.
Combination strings decomposed	1,129	Produced 2,000 component rows.
Components excluded after decomposition	620	Study-scope criteria re-applied to individual components.
Distinct database-by-cleaned-ingredient rows after alias consolidation	3,468	After alias resolution and within-source merging.
Excluded after RxNorm/ATC resolution	168	Diagnostic and other non-therapeutic entries removed after terminology resolution.
Post-resolution salt/synonym consolidations	206	Merged equivalent resolved ingredient representations.
Final harmonized database-by-ingredient rows	3,094	MarketScan 961; INPC 1,051; OSUMC 1,082.
RxNorm-resolved database-by-ingredient rows	3,085	MarketScan 960; INPC 1,049; OSUMC 1,076 (99.7%).

Step	n	Note
ATC-mapped database-by-ingredient rows	2,896	MarketScan 906; INPC 980; OSUMC 1,010 (93.9% of RxNorm-resolved rows).
Distinct RxNorm ingredient concepts across sources	1,315	Deduplicated union across the three sources.
Distinct ATC-mapped ingredient concepts across sources	1,223	Deduplicated union across the three sources.

Panel C. RxNorm resolution and ATC assignment by source

Metric	MarketScan	INPC	OSUMC	Pooled
Ingredients submitted to RxNorm, n	961	1,051	1,082	3,094
RxNorm-resolved ingredients, n	960	1,049	1,076	3,085
Unresolved ingredients, n	1	2	6	9
RxNorm resolution rate (%)	99.9	99.8	99.4	99.7
RxNorm-resolved ingredients with an ATC code, n	906	980	1,010	2,896
RxNorm-resolved ingredients without an ATC code, n	54	69	66	189
ATC assignment rate among RxNorm-resolved ingredients (%)	94.4	93.4	93.9	93.9

Note: Reconciliation counts describe different harmonization stages and are not strictly additive because combination products were decomposed and equivalent ingredient representations were subsequently merged. Pooled mapping values sum database-by-ingredient rows across sources.

Supplementary Table S3. Medication study-scope exclusion and retention criteria.

Panel A. Study-scope exclusion criteria

#	Exclusion criterion	Definition	MarketScan	INPC	OSUMC	Total
1	Devices, supplies, and kits	Devices, consumables, containers, and kits; identifiable therapeutic ingredients were retained.	38	43	111	192
2	Irrigation fluids and intravenous vehicles	Plain irrigation, flush, and vehicle products not representing the therapeutic ingredient of interest.	3	11	29	43
3	Inert excipients, vehicles, and colorants	Pharmacologically inactive formulation components, bases, vehicles, and color additives.	22	34	35	91
4	Nonspecific labels and non-ingredient fragments	Class labels, placeholders, fragments, and bare element names that did not identify a therapeutic ingredient.	38	45	77	160
5	Vaccines and diagnostic preparations	Vaccines, toxoids, diagnostic allergens, contrast media, diagnostic radiopharmaceuticals, and ophthalmic diagnostic agents.	34	63	114	211
6	Nutritional supplements and iron products	Nutritional vitamin/mineral supplements and iron products; prespecified therapeutic substances were retained (Panel B).	78	96	125	299
7	Prenatal and multivitamin products	Prenatal and multivitamin bundles were excluded rather than assigning product counts to individual constituents.	168	25	95	288
8	Herbals, botanicals, and probiotics	Botanical preparations, probiotics, and related non-prespecified supplements.	29	34	40	103
9	Residual non-therapeutic entries	Remaining non-therapeutic products or vocabulary artifacts after normalization.	73	83	94	250
	Total		483	434	720	1,637

Panel B. Prespecified therapeutic substances retained under criterion 6

n	Retention group	Retained substances
17	Therapeutic vitamins and vitamin analogues	alfacalcidol, calcifediol, calcitriol, cyanocobalamin, doxercalciferol, folic acid, folinic acid, hydroxocobalamin, leucovorin, levomefolate, mecobalamin, niacin, paricalcitol, phytonadione, pyridoxal phosphate, pyridoxine, thiamine
31	Therapeutic minerals, electrolytes, and related salts	aluminum hydroxide, ammonium lactate, calcium acetate, calcium chloride, calcium gluconate, calcium polycarbophil, citric acid, ferric subsulfate, magnesium citrate, magnesium hydroxide, magnesium sulfate, magnesium trisilicate, patiromer, potassium acetate, potassium bicarbonate, potassium chloride, potassium citrate, potassium iodide, potassium phosphate, selenium sulfide, silver nitrate, sodium bicarbonate, sodium chloride, sodium citrate, sodium fluoride, sodium hypochlorite, sodium phosphate, sodium polystyrene sulfonate, sodium salicylate, sodium sulfate, sodium zirconium cyclosilicate
2	Prescription omega-3 products	icosapent ethyl, omega-3-acid ethyl esters

Note: Retention in Panel B applied only to criterion 6 and did not override other study-scope exclusions. Nutritional iron products and generic nutritional/fish-oil omega-3 supplements were excluded; prescription omega-3 products were retained.

Supplementary Table S4. Multiple ATC classifications, primary-code assignment, and sensitivity analyses.

Among 1,223 ATC-mapped ingredients, 227 (18.6%) had more than one candidate ATC code. A single primary code was selected using source formulation or route, RxNorm dose form, U.S. product availability, or a prespecified assignment when route or indication could not be distinguished. Detailed ingredient-level assignment records were retained in the harmonization audit file.

Panel A. Earliest ATC level at which candidate codes differed

First divergence level	Ingredients, n	% of multiply classified ingredients
Level 1	184	81.1
Level 2	20	8.8
Level 3	14	6.2
Level 4	8	3.5
Level 5	1	0.4
Total	227	100.0

Panel B. Basis for selecting the primary ATC code

Selection basis	Ingredients, n	% of multiply classified ingredients
Prespecified primary ATC assignment	66	29.1
Source formulation/route	64	28.2
U.S. product availability	41	18.1
RxNorm dose form	33	14.5
Source context + prespecified assignment	16	7.0
Candidates identical through Level 3	7	3.1
Total	227	100.0

Panel C. Level-specific sensitivity analysis

ATC level	Spearman ρ range: primary vs reduced category rankings
Level 1	0.947-0.956
Level 2	0.845-0.862
Level 3	0.848-0.858

Panel D. Conservative sensitivity analysis excluding all multiply classified ingredients

Comparison	Primary Spearman ρ	Reduced Spearman ρ	Absolute change
MarketScan vs INPC	0.793	0.775	0.018
MarketScan vs OSUMC	0.676	0.645	0.031
INPC vs OSUMC	0.800	0.772	0.028

Note: The conservative analysis excluded all ingredients with more than one full ATC code (165 of 759 ingredients shared across all three sources; 21.7%); the largest absolute change in pairwise Spearman correlation was 0.031

Supplementary Table S5. Shared RxNorm-resolved ingredients and Jaccard similarity across rank thresholds.

At each threshold, ingredients were ranked by standardized utilization within each source. Jaccard similarity = intersection / union for each source pair.

Top N	Shared by all three	MarketScan vs INPC (n)	MarketScan vs INPC (Jaccard)	MarketScan vs OSUMC (n)	MarketScan vs OSUMC (Jaccard)	INPC vs OSUMC (n)	INPC vs OSUMC (Jaccard)
10	2	7	0.538	2	0.111	4	0.250
20	2	12	0.429	2	0.053	8	0.250
50	9	29	0.408	11	0.124	24	0.316
100	44	68	0.515	46	0.299	67	0.504
150	83	108	0.562	87	0.408	115	0.622
200	118	150	0.600	125	0.455	159	0.660
250	158	189	0.608	167	0.502	204	0.689
300	193	232	0.630	204	0.515	244	0.685
350	234	276	0.651	244	0.535	284	0.683
400	269	319	0.663	283	0.547	322	0.674
450	306	361	0.670	326	0.568	362	0.673
500	339	398	0.661	366	0.577	399	0.664
550	375	443	0.674	400	0.571	437	0.659
600	416	484	0.676	451	0.602	481	0.669
650	454	528	0.684	492	0.609	529	0.686
700	486	563	0.673	534	0.617	564	0.675
750	520	601	0.669	587	0.643	599	0.665
800	554	634	0.656	629	0.648	642	0.670
850	591	673	0.655	677	0.662	681	0.668
900	642	726	0.676	720	0.667	727	0.678
950	679	773	0.686	758	0.664	771	0.683
All resolved ingredients	759	830	0.704	822	0.677	877	0.703

Supplementary Table S6. Cumulative share of standardized medication utilization accounted for by the top ATC Level-2 classes in each source.

Top N Level-2 classes	MarketScan (%)	INPC (%)	OSUMC (%)
10	64.46	60.61	69.48
20	86.66	82.39	89.37
30	95.08	93.27	95.81
40	97.96	98.07	98.70
50	99.33	99.43	99.65

Number of Level-2 classes observed: MarketScan 78; INPC 80; OSUMC 81.

Supplementary Table S7. ATC Level-3 utilization distribution, cumulative share, and pairwise correlations.

Panel A. Twenty Level-3 classes with the highest pooled utilization (classes shown in Supplementary Figure S2), ordered by largest source share

ATC Level-3	Class	MarketScan (%)	INPC (%)	OSUMC (%)
N01B	Local Anesthetics	0.32	2.74	9.54
A06A	Drugs for Constipation	0.23	1.97	8.43
G02A	Uterotonics	0.04	1.81	6.93
J01X	Other Antibacterials	6.79	5.80	1.02
R06A	Antihistamines for Systemic Use	5.99	5.78	4.17
J01C	Beta-Lactam Antibacterials, Penicillins	5.86	4.64	0.87
N02B	Other Analgesics and Antipyretics	3.03	5.70	5.04
B05X	I.V. Solution Additives	0.03	3.71	5.63
J01F	Macrolides, Lincosamides and Streptogramins	5.37	3.30	0.83
H01B	Posterior Pituitary Lobe Hormones	0.01	2.56	5.31
N02A	Opioids	0.82	2.44	5.21
A04A	Antiemetics and Antinauseants	5.20	4.11	4.94
G03D	Progestogen Sex Hormones and Modulators of the Genital System	4.56	1.67	0.49
M01A	Antiinflammatory and Antirheumatic Products, Non-Steroids	1.07	1.17	4.22
J01D	Other Beta-Lactam Antibacterials	3.07	3.69	1.74
N06A	Antidepressants	3.65	2.54	0.78
J05A	Direct Acting Antivirals	3.62	1.31	0.31
R01A	Decongestants and Other Nasal Preparations for Topical Use	1.50	3.30	0.99
A02B	Drugs for Peptic Ulcer and Gastro-Oesophageal Reflux Disease (Gord)	2.70	2.98	1.36
H02A	Corticosteroids for Systemic Use, Plain	2.40	2.04	1.00

Panel B. Cumulative share of the top Level-3 classes ranked within each source

Top N Level-3 classes	MarketScan (%)	INPC (%)	OSUMC (%)
10	47.13	43.02	59.42
20	71.41	66.04	79.38
30	84.55	79.10	88.27
40	91.21	87.93	93.01
50	95.06	93.74	96.00

Panel C. Pairwise correlations of Level-3 shares among classes present in both sources

Comparison	Shared classes	Pearson r	Spearman ρ	Kendall τ
MarketScan vs INPC	159	0.812	0.865	0.706
MarketScan vs OSUMC	159	0.216	0.709	0.531
INPC vs OSUMC	163	0.604	0.892	0.716

Supplementary Table S8. Top 50 RxNorm ingredients unique to each data source.

Within each source, the 50 database-specific ingredients with the highest standardized utilization rates are shown, ordered by rate.

Data source	RxNorm ingredient	Rate per 1,000	Primary ATC code
MarketScan	eicosapentaenoate	0.2413	-
MarketScan	oxyquinoline	0.0233	G01AC30
MarketScan	nedocromil	0.0137	S01GX04
MarketScan	dexchlorpheniramine	0.0123	R06AB02
MarketScan	alosetron	0.0110	A03AE01
MarketScan	clioquinol	0.0096	D08AH30
MarketScan	dexbrompheniramine	0.0096	R06AB06
MarketScan	chlophedianol	0.0082	R05DB10
MarketScan	azilsartan	0.0055	C09CA09
MarketScan	iloperidone	0.0055	N05AX14
MarketScan	octinoxate	0.0055	D02BA02
MarketScan	betaxolol	0.0041	S01ED02
MarketScan	imiglucerase	0.0041	A16AB02
MarketScan	ocrelizumab	0.0041	L04AG08
MarketScan	palivizumab	0.0041	J06BD01
MarketScan	policosanol	0.0041	C10AX08
MarketScan	sacrosidase	0.0041	A16AB06
MarketScan	silodosin	0.0041	G04CA04
MarketScan	alpha 1-antitrypsin	0.0027	B02AB02
MarketScan	auranofin	0.0027	M01CB03
MarketScan	carteolol	0.0027	S01ED05
MarketScan	ceftibuten	0.0027	J01DD14
MarketScan	danazol	0.0027	G03XA01
MarketScan	droxidopa	0.0027	C01CA27
MarketScan	ecallantide	0.0027	B06AC03
MarketScan	estropipate	0.0027	-
MarketScan	flutamide	0.0027	L02BB01
MarketScan	hydroxypropylcellulose	0.0027	-
MarketScan	methazolamide	0.0027	S01EC05
MarketScan	nafarelin	0.0027	H01CA02
MarketScan	tegaserod	0.0027	A06AX06
MarketScan	telbivudine	0.0027	J05AF11
MarketScan	trandolapril	0.0027	C09AA10
MarketScan	tranylcypromine	0.0027	N06AF04
MarketScan	5-methyltetrahydrofolic acid	0.0014	-
MarketScan	alectinib	0.0014	L01ED03
MarketScan	alemtuzumab	0.0014	L04AG06
MarketScan	avanafil	0.0014	G04BE10
MarketScan	becaplermin	0.0014	D03AX06
MarketScan	benzphetamine	0.0014	-
MarketScan	brodalumab	0.0014	L04AC12
MarketScan	chlorpropamide	0.0014	A10BB02
MarketScan	cysteamine	0.0014	A16AA04
MarketScan	deutetrabenazine	0.0014	N07XX16
MarketScan	elbasvir	0.0014	J05AP10
MarketScan	estazolam	0.0014	N05CD04
MarketScan	etoposide	0.0014	L01CB01
MarketScan	fosamprenavir	0.0014	J05AE07
MarketScan	grazoprevir	0.0014	J05AP11
MarketScan	lanadelumab	0.0014	B06AC05

Supplementary Table S8. Top 50 RxNorm ingredients unique to each data source (continued).

Data source	RxNorm ingredient	Rate per 1,000	Primary ATC code
INPC	dolasetron	0.5051	A04AA04
INPC	magnesium phosphate	0.5007	B05XA10
INPC	remdesivir	0.2083	J05AB16
INPC	thiopental	0.1905	N01AF03
INPC	potassium acetate	0.1684	B05XA17
INPC	nelfinavir	0.1595	J05AE04
INPC	chloroxylenol	0.1507	D08AE05
INPC	phenylpropanolamine	0.1507	R01BA01
INPC	terfenadine	0.1507	R06AX12
INPC	radium	0.1285	-
INPC	kaolin	0.1152	A07BC02
INPC	casanthranol	0.1063	-
INPC	metocurine	0.0886	-
INPC	thiethylperazine	0.0798	R06AD03
INPC	ritodrine	0.0665	G02CA01
INPC	carbenicillin	0.0620	J01CA03
INPC	lysine	0.0576	B05XB03
INPC	choline	0.0443	N07AX02
INPC	cortisone	0.0443	H02AB10
INPC	resorcinol	0.0443	D10AX02
INPC	sufentanil	0.0443	N01AH03
INPC	alfentanil	0.0222	N01AH02
INPC	anti-inhibitor coagulant complex	0.0222	B02BD01
INPC	astemizole	0.0222	R06AX11
INPC	attapulgite	0.0222	A07BC04
INPC	bretylium	0.0222	C01BD02
INPC	brigatinib	0.0222	L01ED04
INPC	calcium glycerophosphate	0.0222	-
INPC	Capsicum extract	0.0222	-
INPC	cefditoren	0.0222	J01DD16
INPC	ceftaroline fosamil	0.0222	J01DI02
INPC	ceritinib	0.0222	L01ED02
INPC	chlorothiazide	0.0222	C03AA04
INPC	cisapride	0.0222	A03FA02
INPC	cisplatin	0.0222	L01XA01
INPC	collagen	0.0222	G04BX11
INPC	crizanlizumab	0.0222	B06AX01
INPC	dacarbazine	0.0222	L01AX04
INPC	dalbavancin	0.0222	J01XA04
INPC	deferiprone	0.0222	V03AC02
INPC	deoxyribonucleases	0.0222	B06AA10
INPC	diazoxide	0.0222	V03AH01
INPC	didanosine	0.0222	J05AF02
INPC	dienestrol	0.0222	G03CB01
INPC	doxercalciferol	0.0222	H05BX03
INPC	ethacrynate	0.0222	C03CC01
INPC	ezogabine	0.0222	N03AX21
INPC	fibrinogen	0.0222	B02BB01
INPC	fosaprepitant	0.0222	-
INPC	gemcitabine	0.0222	L01BC05

Supplementary Table S8. Top 50 RxNorm ingredients unique to each data source (continued).

Data source	RxNorm ingredient	Rate per 1,000	Primary ATC code
OSUMC	nirmatrelvir	0.7878	-
OSUMC	sevoflurane	0.4115	N01AB08
OSUMC	desflurane	0.1756	N01AB07
OSUMC	hetastarch	0.1229	B05AA07
OSUMC	dactinomycin	0.1004	L01DA01
OSUMC	zuranolone	0.0803	N06AX31
OSUMC	sodium zirconium cyclosilicate	0.0577	V03AE10
OSUMC	enalaprilat	0.0477	C09AA02
OSUMC	bevacizumab	0.0452	L01FG01
OSUMC	goserelin	0.0427	L02AE03
OSUMC	benzethonium	0.0326	R02AA09
OSUMC	aflibercept	0.0301	L01XX44
OSUMC	pyrethrins	0.0301	-
OSUMC	zoledronic acid	0.0301	M05BA08
OSUMC	lenalidomide	0.0276	L04AX04
OSUMC	denosumab	0.0251	M05BX04
OSUMC	pyrilamine	0.0251	R06AC01
OSUMC	cenobamate	0.0201	N03AX25
OSUMC	antithrombin III	0.0176	B01AB02
OSUMC	letermovir	0.0176	J05AX18
OSUMC	beta carotene	0.0151	A11CA02
OSUMC	epcoritamab	0.0151	L01FX27
OSUMC	paricalcitol	0.0151	H05BX02
OSUMC	trypan blue	0.0151	S01KX02
OSUMC	argatroban	0.0125	B01AE03
OSUMC	cefotaxime	0.0125	J01DD01
OSUMC	isoflurane	0.0125	N01AB06
OSUMC	ofatumumab	0.0125	L04AG12
OSUMC	temozolomide	0.0125	L01AX03
OSUMC	cetylpyridinium	0.0100	R02AA06
OSUMC	clevidipine	0.0100	C08CA16
OSUMC	doravirine	0.0100	J05AG06
OSUMC	doxapram	0.0100	R07AB01
OSUMC	isavuconazonium	0.0100	J02AC05
OSUMC	treprostinil	0.0100	B01AC21
OSUMC	voxelotor	0.0100	B06AX03
OSUMC	alfuzosin	0.0075	G04CA01
OSUMC	atogepant	0.0075	N02CD07
OSUMC	dotatate	0.0075	V10XX04
OSUMC	factor XIII	0.0075	B02BD07
OSUMC	finerenone	0.0075	C03DA05
OSUMC	fulvestrant	0.0075	L02BA03
OSUMC	lumateperone	0.0075	N05AD10
OSUMC	metreleptin	0.0075	A16AA07
OSUMC	tenecteplase	0.0075	B01AD11
OSUMC	tromethamine	0.0075	B05BB03
OSUMC	ambrisentan	0.0050	C02KX02
OSUMC	amifampridine	0.0050	N07XX05
OSUMC	bicalutamide	0.0050	L02BB03
OSUMC	brilliant blue G	0.0050	-

Note: Database-specific indicates that an ingredient was observed in only one source after harmonization; it does not imply pregnancy-specific use or greater clinical relevance.
Ingredients with equal rates are listed alphabetically, so the cut-off at 50 may fall within a group of tied values. In INPC, a rate of 0.0222 per 1,000 corresponds to a privacy-suppressed count (<10) imputed as 5.

Supplementary Table S9. ATC Level-2 source enrichment and sensitivity to the leading-source margin threshold.

Leading-source margin = highest source share − second-highest source share. Enrichment = highest source share − mean of the other two source shares.

Panel A. Level-2 classes meeting the 1.5-percentage-point leading-source margin threshold

Rank	ATC Level-2	Class	MarketScan (%)	INPC (%)	OSUMC (%)	Leading source	Leading-source margin (pp)	Enrichment (pp)
1	N01	Anesthetics	0.32	4.92	11.86	OSUMC	6.94	9.24
2	A06	Drugs for Constipation	0.23	1.97	8.43	OSUMC	6.46	7.33
3	G03	Sex Hormones and Modulators of the Genital System	8.35	3.03	0.99	MarketScan	5.33	6.34
4	G02	Other Gynecologicals	0.14	1.83	6.93	OSUMC	5.10	5.94
5	B05	Blood Substitutes and Perfusion Solutions	0.07	4.13	8.31	OSUMC	4.18	6.21
6	J01	Antibacterials for Systemic Use	23.19	19.03	4.95	MarketScan	4.16	11.20
7	M01	Antiinflammatory and Antirheumatic Products	1.07	1.17	4.22	OSUMC	3.05	3.10
8	H01	Pituitary and Hypothalamic Hormones and Analogues	0.10	2.58	5.32	OSUMC	2.74	3.98
9	R01	Nasal Preparations	1.60	3.96	1.03	INPC	2.36	2.65
10	J05	Antivirals for Systemic Use	3.62	1.31	0.31	MarketScan	2.30	2.80
11	N02	Analgesics	4.15	8.29	10.33	OSUMC	2.03	4.10
12	H03	Thyroid Therapy Drugs	2.90	0.88	0.25	MarketScan	2.03	2.34
13	A11	Vitamins	2.94	1.28	0.47	MarketScan	1.66	2.07
14	N06	Psychoanaleptics	5.25	3.64	0.88	MarketScan	1.61	2.99
15	A03	Drugs for Functional Gastrointestinal Disorders	1.38	1.14	2.94	OSUMC	1.57	1.69

Panel B. Classes retained at alternative leading-source margin thresholds

Threshold (pp)	Classes retained	MarketScan	INPC	OSUMC
0.25	32	16	4	12
0.5	24	9	3	12
0.75	21	8	2	11
1	19	8	1	10
1.25	16	6	1	9
1.5	15	6	1	8
2	12	4	1	7
2.5	8	2	0	6
3	7	2	0	5
4	6	2	0	4
5	4	1	0	3

Supplementary Figure S1. Shared medications and Jaccard similarity by rank threshold.

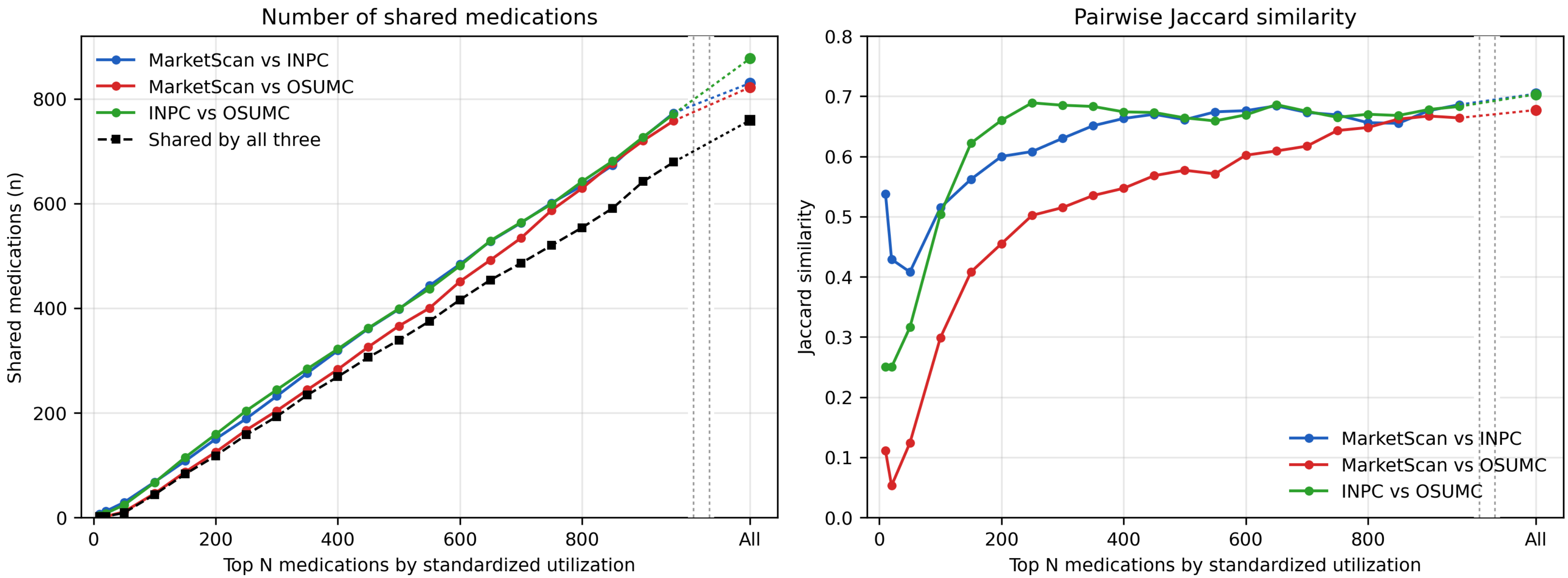

Note: Medications were ranked within each database by standardized utilization. The left panel shows pairwise counts of shared medications and the number shared by all three sources at each top-N threshold; the right panel shows pairwise Jaccard similarity (intersection/union). The final point represents all RxNorm-resolved ingredients.

Supplementary Figure S2. Top 20 pooled ATC Level-3 subgroups.

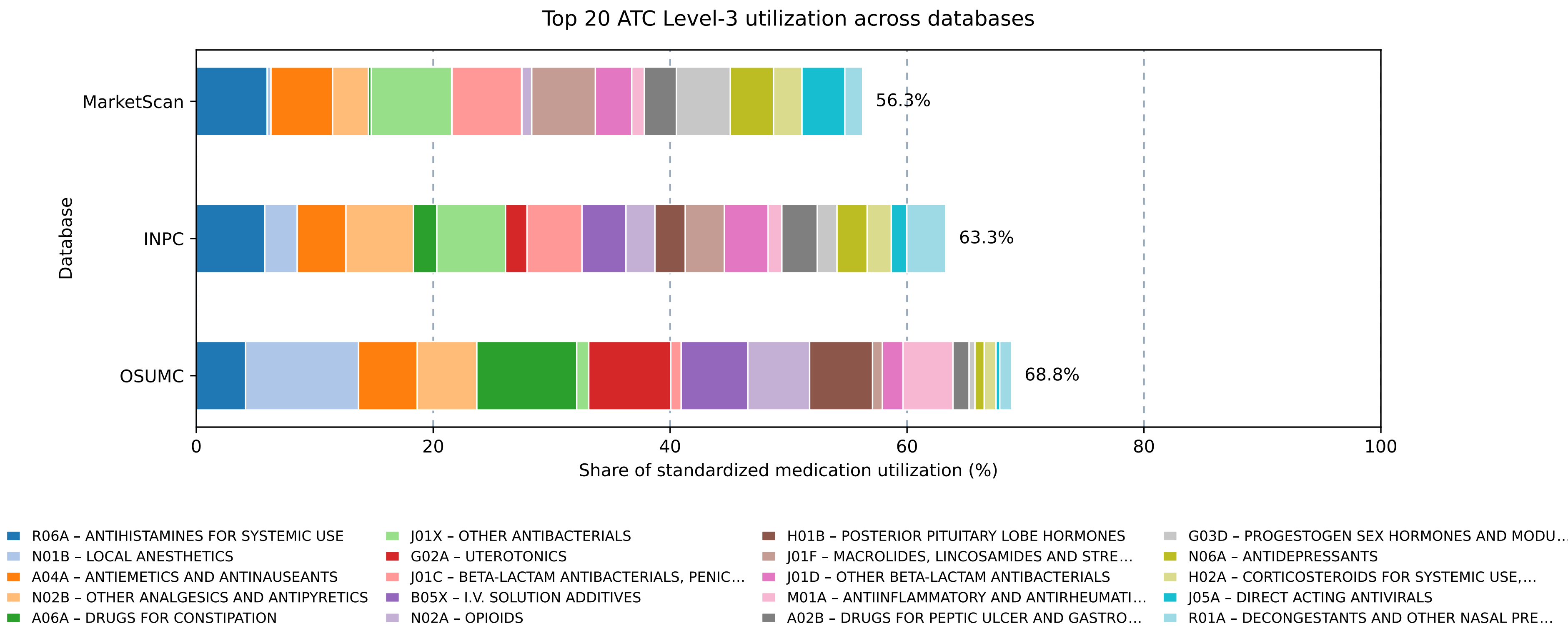

Note: Stacked bars show the contribution of the 20 displayed ATC Level-3 subgroups to standardized medication utilization within each database. Percent labels indicate the cumulative share accounted for by these 20 subgroups. ATC, Anatomical Therapeutic Chemical Classification System; INPC, Indiana Network for Patient Care; OSUMC, The Ohio State University Medical Center.

Supplementary Figure S3. ATC Level-1 profile of database-specific medications.

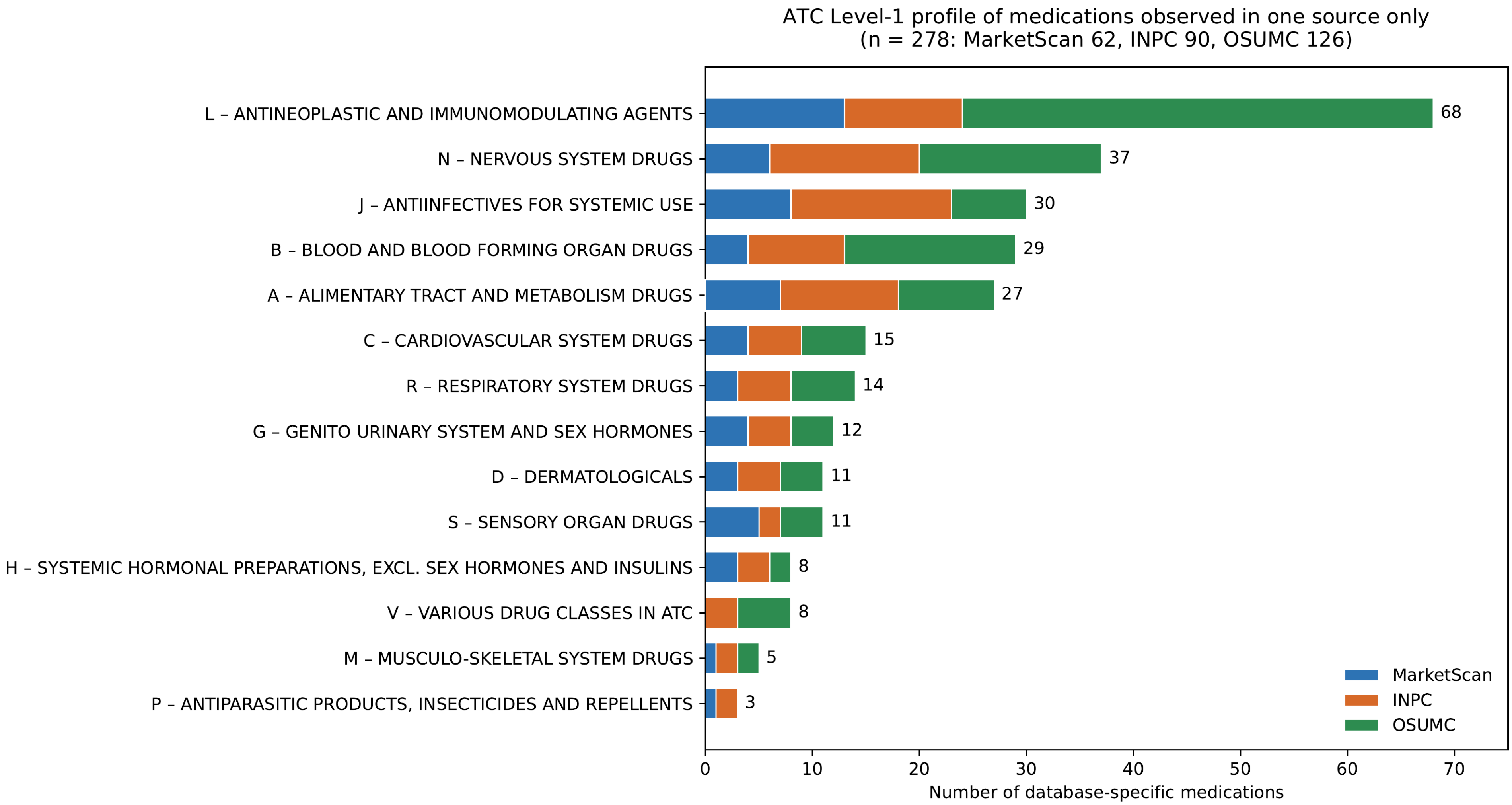

Note: Bars show harmonized medications observed in only one data source, grouped by primary ATC Level-1 category. Numbers at the ends of bars indicate total counts across the three sources. ATC, Anatomical Therapeutic Chemical Classification System; INPC, Indiana Network for Patient Care; OSUMC, The Ohio State University Medical Center.
